# Extinction-Dependent Alterations in Corticostriatal mGluR2/3 and mGluR7 Receptors following Chronic Methamphetamine Self-Administration in Rats

**DOI:** 10.1371/journal.pone.0034299

**Published:** 2012-03-29

**Authors:** Marek Schwendt, Carmela M. Reichel, Ronald E. See

**Affiliations:** Department of Neurosciences, Medical University of South Carolina, Charleston, South Carolina, United States of America; Chiba University Center for Forensic Mental Health, Japan

## Abstract

Methamphetamine (meth) is a highly addictive and widely abused psychostimulant. Repeated use of meth can quickly lead to dependence, and may be accompanied by a variety of persistent psychiatric symptoms and cognitive impairments. The neuroadaptations underlying motivational and cognitive deficits produced by chronic meth intake remain poorly understood. Altered glutamate neurotransmission within the prefrontal cortex (PFC) and striatum has been linked to both persistent drug-seeking and cognitive dysfunction. Therefore, the current study investigated changes in presynaptic mGluR receptors within corticostriatal circuitry after extended meth self-administration. Rats self-administered meth (or received yoked-saline) in 1 hr/day sessions for 7 days (short-access) followed by 14 days of 6 hrs/day (long-access). Rats displayed a progressive escalation of daily meth intake up to 6 mg/kg per day. After cessation of meth self-administration, rats underwent daily extinction or abstinence without extinction training for 14 days before being euthanized. Synaptosomes from the medial PFC, nucleus accumbens (NAc), and the dorsal striatum (dSTR) were isolated and labeled with membrane-impermeable biotin in order to measure surface mGluR2/3 and mGluR7 receptors. Extended access to meth self-administration followed by abstinence decreased surface and total levels of mGluR2/3 receptors in the NAc and dSTR, while in the PFC, only a loss of surface mGluR2/3 and mGluR7 receptors was detected. Daily extinction trials reversed the downregulation of mGluR2/3 receptors in the NAc and dSTR and mGluR7 in the PFC, but downregulation of surface mGluR2/3 receptors in the PFC was present regardless of post-meth experience. Thus, extinction learning can selectively restore some populations of downregulated mGluRs after prolonged exposure to meth. The present findings could have implications for our understanding of the persistence (or recovery) of meth-induced motivational and cognitive deficits.

## Introduction

Methamphetamine (meth) is a widely abused and highly addictive psychostimulant. While acute meth produces short-term ‘positive’ subjective effects and increased psychomotor/cognitive performance [1], continued abuse often leads to compulsive drug taking, addiction, and long-term deleterious health consequences. In addition to meth-induced peripheral pathologies (e.g., weight loss, cardiovascular toxicity, and severe tooth decay), chronic meth use can result in a variety of psychiatric symptoms and cognitive impairments. Psychosis, attention and memory deficits, impulsivity, and increased anxiety and aggression have been documented in active and abstinent meth addicts [2], [3], [4], [5], [6]. Clinical studies consistently demonstrate that chronic meth users have high rates of relapse that are equal to, if not higher than, drugs such as cocaine and heroin [7]. Despite the fact that meth represents a serious health concern, cognitive behavioral therapy constitutes the only treatment option [8], [9] as no approved pharmacotherapies exist for the treatment of meth addiction and its neuropsychological consequences [10].

Our limited understanding of chronic meth-induced neuroadaptations in humans or experimental animals has impeded the development of successful meth addiction treatment. Rodent models of extended daily access to meth self-administration are highly suitable for identifying such plasticities, as they possess good face validity for meth addiction in humans. As such, rats with extended daily access to intravenous meth typically display escalation of meth-intake [11], [12], [13] and enhanced drug-seeking [12], [13] when compared to more limited-access conditions. In addition, extended meth access in rats results in lasting cognitive impairments, specifically in attention and memory domains [12], [14], similar to those observed in a significant portion of meth addicts [2].

Meth rapidly increases extracellular levels of monoamines, enhancing dopamine, norepinephrine, and serotonin release [4], [15]. In addition to monoamines, acute meth exposure increases extracellular glutamate in several brain regions, including the frontal cortex, hippocampus, dorsal striatum, nucleus accumbens, and the ventral tegmental area (for review see: [16]). Previous research has largely focused on the role of glutamate in neurotoxic damage produced by acute high doses of experimenter-administered meth [17], [18]. Under these conditions, excessive and prolonged glutamate release in the striatum and frontal cortex is typically observed. However, when meth delivery occurs at lower doses and/or under contingent conditions, glutamate neurotransmission likely plays a key role in mediating rewarding and reinforcing effects of meth [19], [20]. In support of this, systemic blockade of NMDA or mGluR5 glutamate receptors attenuated meth self-administration [19], [21], [22] and blocked the reinstatement of meth-seeking behavior [19]. In a recent study [14], we showed that systemic allosteric modulation of mGluR5 receptors can reverse deficits in recognition memory caused by extended meth self-administration, suggesting that dysregulated glutamate neurotransmission underlies some facets of the cognitive deficits seen in meth addiction.

In order to further investigate chronic meth-induced glutamatergic abnormalities, the current study analyzed changes in the number of cell-surface (functional) mGluR2/3 and mGluR7 receptors in the medial prefrontal cortex (PFC) and the striatum as a result of extended meth self-administration followed by a drug-free abstinence period or daily extinction trials. We chose these regional receptor populations based on evidence showing that: (1) Glutamatergic input from the PFC into the striatum plays a critical role in regulating drug-seeking [23], [24], [25] and certain types of recognition memory [26], (2) mGluR2/3 and mGluR7 are highly enriched in corticostriatal projection neurons, acting as autoreceptors in glutamatergic terminals that modulate glutamate homeostasis during abstinence and reinstatement of drug-seeking [25], [27], and (3) extended psychostimulant induced changes in mGluR2/3 (and possibly mGluR7) receptor sensitivity in the corticostriatal circuitry has been postulated as a critical neuroadaptation linked to increased relapse vulnerability [27], [28], [29].

## Materials and Methods

### Subjects

Male Long-Evans rats (Charles River Laboratories, Wilmington, MA) weighing 275–300 g at the time of delivery were individually housed in a temperature- and humidity-controlled vivarium on a reversed 12 h light-dark cycle. Rats received *ad libitum* water throughout the study and 25 g of standard rat chow (Harlan, Indianapolis, IN) daily until self-administration stabilized, at which time animals were maintained *ad libitum*. All animal procedures were approved by the Institutional Animal Care and Use Committee of the Medical University of South Carolina and were performed in accordance with the Guide for the Care and Use of Laboratory Animals.

### Catheter surgery

On the day of surgery, rats were anesthetized with ketamine/xylazine (66 mg/kg and 1.33 mg/kg i.p.), followed by Equithesin (0.5 ml/kg i.p.). In addition, Ketorolac (2.0 mg/kg, i.p.) was given preoperatively as an analgesic. During anesthesia, all animals were implanted with indwelling catheters placed into the right jugular vein. Catheter construction and surgical procedures were previously described [14], [30]. An antibiotic solution of cefazolin (10 mg/0.1 ml; Schein Pharmaceuticals, Florham Park, NJ) was given along with 0.1 ml 70 U/ml heparinized saline post surgery and during recovery. During self-administration, rats received an infusion (0.1 ml) of 10 U/ml heparinized saline (Elkins-Sinn, Cherry Hill, NJ) before each session. After each session, catheters were flushed with cefazolin and 70 U/ml heparinized saline. Catheter patency was periodically verified with methohexital sodium (10 mg/ml dissolved in 0.9% physiological saline; Eli Lilly, Indianapolis, IN), a short-acting barbiturate that produces a rapid loss of muscle tone when administered intravenously.

### Methamphetamine self-administration, extinction, and abstinence procedures

Meth self-administration occurred in standard operant conditioning chambers (30×20×20 cm, Med Associates, East Fairfield, VT) housed inside sound-attenuating cubicles containing a fan for airflow and masking noise. The chambers were equipped with a house light, two retractable levers, two stimulus lights, and a tone generator. Additionally, each chamber had a balanced metal arm and spring leash attached to a swivel (Instech, Plymouth Meeting, PA) with Tygon® tubing extending through the leash and connected to a 10 ml syringe mounted on an infusion pump.

**Figure 1:**
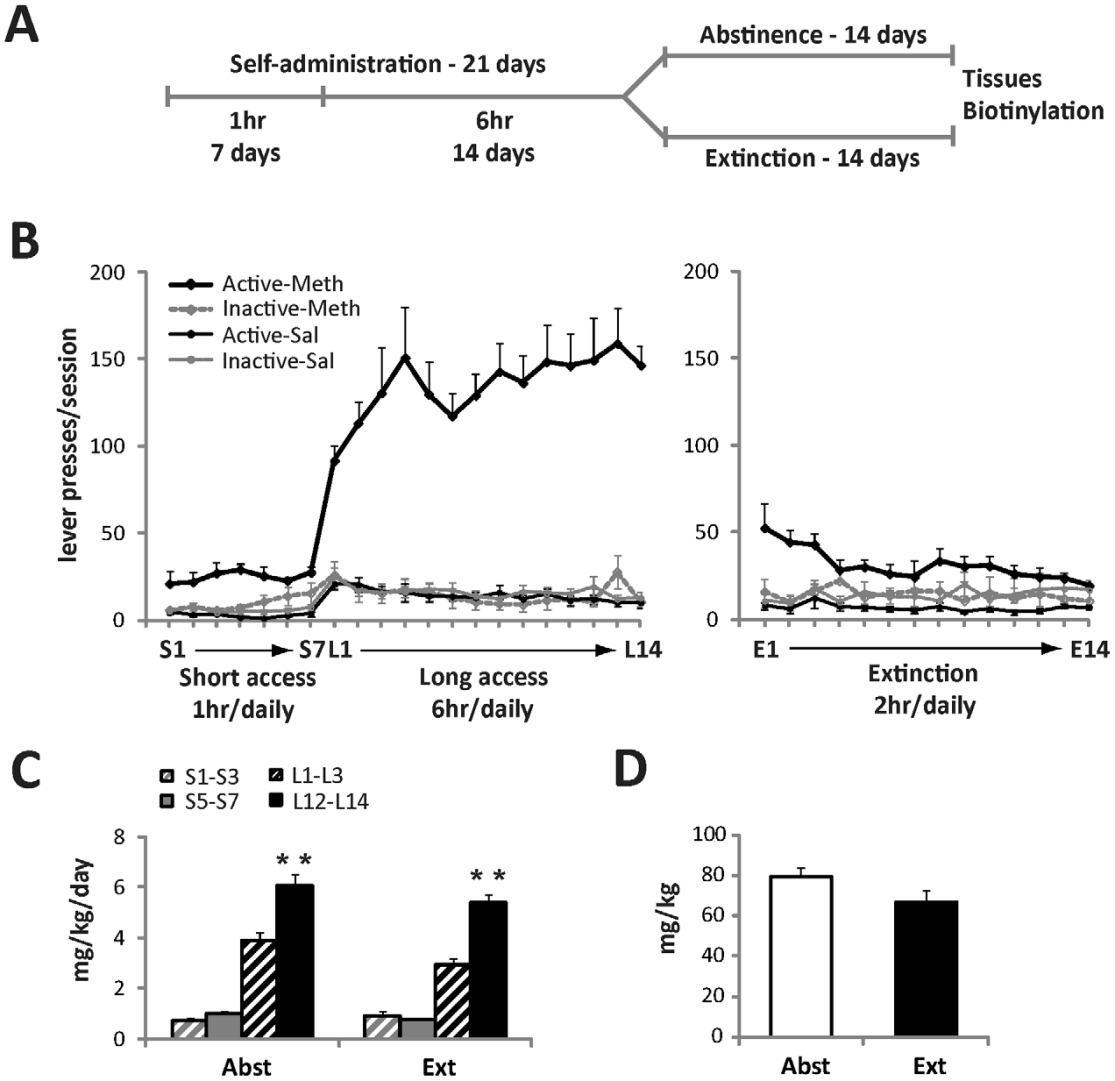
Extended access to intravenous meth results in robust self-administration and escalation of meth intake. (A) Timeline of the experiment as described in detail in the Materials and Methods section. Animals underwent 21 days of meth self-administration (or received yoked-saline) followed by 14 days of home cage abstinence or 14 daily extinction sessions. Tissues were collected at the end of the experiment. (B) Daily lever responding for meth and yoked-sal animals during self-administration (left graph) and extinction (right graph). (C) Escalation of daily meth intake during short (1 hr) and long (6 hr) self-administration sessions as detected in abstinent and extinction animals. Mean daily meth intake over the course of the first three days vs. last three days of short- and long-access self-administration was analyzed by one-way ANOVA and expressed as mg/kg/day of meth. (D) Total meth intake (mg/kg) in abstinent vs. extinction animals. Data shown as mean ± S.E.M.; n = 8–11 samples per group. **p<0.01 L12–L14 vs. L1–L3.

Following at least 5 days of recovery from surgery, rats were randomly assigned to either meth or yoked-saline groups and underwent 21 days of self-administration. As shown in Figure 1A, meth (Sigma, St. Louis, MO; 0.4 mg/ml dissolved in sterile saline) was self-administered in 1 hr/day sessions for 7 days (short-access) followed by 14 days of 6 hr/day (long-access). The house light always signaled the beginning of a session and remained on throughout the session. During the sessions, a response on the active lever resulted in activation of the pump for a 2-sec meth infusion (20 µg/50 µl bolus infusion) and presentation of a 5-sec tone (78 dB, 4.5 kHz) and a white stimulus light over the active lever, followed by a 20-sec time out. Yoked controls received a 50 µl bolus of saline when the matched subject received the contingent meth infusion. Responses occurring during the time out and on the inactive lever were recorded, but had no scheduled consequences. All sessions took place during the dark cycle.

Following self-administration, rats were divided into two groups during the 2 week withdrawal period, whereby one group experienced daily extinction trials and the other group underwent abstinence in the home cage (Fig. 1A). This experimental design was based on previous studies indicating that extinction training vs. abstinence result in distinct neural and behavioral adaptations [31], [32]. During extinction, presses on the previously active lever were recorded but no longer produced drug or presentation of the drug-paired cues (light+tone). In contrast, animals in the abstinence group were transported and handled on a daily basis, but not returned to the operant conditioning chambers.

### Tissue collection and synaptosomal preparation

**Figure 2:**
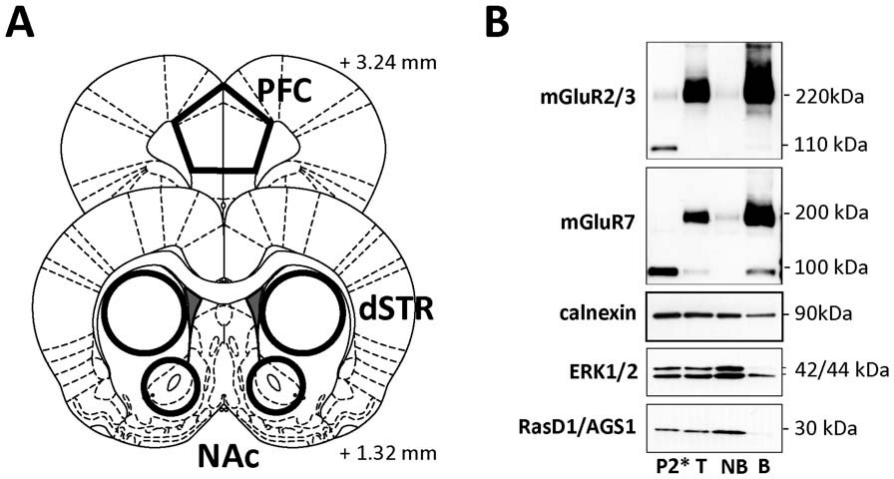
Analysis of mGluR2/3 and mGluR7 surface vs. intracellular distribution in rat brain synaptosomes. (A) Rat brain atlas coordinates (adapted from [77]) used for tissue dissection and synaptosome preparation. (B) Representative immunoblots showing surface vs. intracellular distribution of mGluR2/3 and mGluR7 receptors (as well as marker proteins: calnexin, ERK1/2 and RasD1/AGS1) in synaptosomal fraction prepared from the dSTR. P2* - synaptosomal fraction resolved under strongly reducing conditions (100 mM DTT); T – total synaptosomal fraction after biotinylation resolved under weakly reducing conditions (>10 mM DTT); NB – non-biotinylated (intracellular) proteins; B – biotinylated (surface) proteins isolated by precipitation with streptavidin-agarose beads.

Fourteen days after the last self-administration session for the abstinent group and 22 hr after the 14^th^ day of extinction for the extinction group, rats were rapidly decapitated and brains quickly removed and chilled on ice. Tissues of interest, medial PFC (primarily prelimbic cortex), nucleus accumbens (NAc) and dorsal striatum (dSTR), were hand-dissected from 2–3 mm thick sections (as depicted on Figure 2A) obtained using a Precision brain slicer (Braintree Scientific, Braintree, MA). For preparation of crude synaptosomes, the method published by Samuvel et al. [33] was followed, with minor modifications. Briefly, immediately after dissection, tissues were collected and homogenized with a glass-teflon homogenizer in ∼10 volumes (w/v) of ice-cold 0.32 M sucrose/10 mM HEPES buffer containing the following inhibitors: Complete Mini protease inhibitor (Roche Diagnostics, Indianapolis, IN) and Halt phosphatase inhibitor (Thermo Fisher Scientific, Rockford, IL). Homogenates were first centrifuged at 800 *g* for 10 min at 4°C and the resulting supernatant then centrifuged at 15,000 g for 20 min. The pellet (containing crude synaptosomal membranes) was washed by resuspending in sucrose buffer and protein concentrations were determined by Micro-Bicinchoninic Acid assay according to the manufacturer's instructions (Thermo Fisher Scientific).

### Surface biotinylation and immunoblotting

Synaptosomes (500 µg) were treated with EZlink sulfo-NHS-SS-biotin (1 mg/1 mg protein; Thermo Fisher Scientific) for 1 hr at 4°C in ice-cold PBS/Ca/Mg buffer [137 mM NaCl, 2.7 mM KCl, 4.3 mM, Na_2_HPO_4_, 1.4 mM KH_2_PO_4_, 0.1 mM CaCl_2_, 1 mM MgCl_2_, pH = 7.4]. Subsequently, the samples were washed with the same buffer containing 100 mM glycine to quench biotinylation reaction and rapidly pelleted by centrifugation. The pellet was then resuspended by sonication in RIPA lysis buffer [50 mM Tris HCl pH 7.6, 150 mM NaCl, 1% NP-40, 0.5% sodium deoxycholate, 0.1% SDS] supplemented with protease inhibitors and phosphatase inhibitors (as described above). Following incubation for 1 hr at 4°C, insoluble debris was removed by centrifugation at 24,000 *g* for 10 min and an aliquot of precleared solubilizate (TOTAL protein) was saved. Biotinylated proteins were isolated by incubating with streptavidin agarose beads (Thermo Fisher Scientific) overnight at 4°C. After a brief centrifugation, supernatant (unbound, NON-BIOTINYLATED protein fraction) was saved and beads were washed three times with RIPA assay buffer. Bound (BIOTINYLATED) proteins were eluted with Laemmli sample buffer (62.5 mM Tris-HCl, pH 6.8, 20% glycerol, 2% SDS, and 100 mM DTT) for 30 min at 37°C. Aliquots from TOTAL extracts (5 µg), NON-BIOTINYLATED fractions (5 µg), and 15 µl of eluted BIOTINYLATED fractions were separated by SDS-PAGE (4–15%), transferred to PVDF membrane. Membranes were blocked for 1 hr in 5% milk/Tris-buffered saline and probed overnight at 4°C with primary antibodies diluted in 5% milk/Tris-buffered saline with 0.1% Tween 20. The following primary antisera were used: anti-mGluR2/3 and anti-mGluR7 (Millipore, Billerica, MA), anti-total ERK1/2 and anti-total Akt (Cell Signalling Technology, Danvers, MA), anti-RasD1/AGS1 [34] and anti-calnexin (Enzo Life Sciences, Farmingdale, NY). After incubation with an appropriate HRP-conjugated secondary antiserum (Jackson Immuno Research, West Grove, PA), immunoreactive bands on the membranes were detected by ECL+ chemiluminescence reagents on an X-ray film (GE Healthcare, Piscataway, NJ). Subsequently, the blots were stripped and reprobed with anti-RasD1/DexRas1 antibody to monitor biotinylation of intracellular proteins and with anti-calnexin antibody to normalize for unequal loading and/or transfer of proteins. The integrated band density of each protein sample was measured using NIH Image J software version 1.32j (http://rsb.info.nih.gov/ij/).

### Statistical analysis

Meth intake (mg/kg) was calculated for the final 3 days of intake and compared between the two meth groups using a Student's *t*-test. Immunoblotting data, represented by integrated density of individual bands, were normalized for the density of calnexin immunoreactivity within the same sample, analyzed by a Student's *t*-test, and expressed as the percentage of the saline-treated animals in total or biotinylated samples. Sigma Stat (SPSS, Chicago, IL) software was used for statistical analysis of all data. A value of *p*<0.05 was considered statistically significant.

## Results

### Extended daily meth access results in robust self-administration and escalation of meth intake

All animals self-administered meth, or received yoked saline infusions, during 21 daily sessions. As shown on Figure 1B (left panel), meth rats quickly learned to distinguish between the active and inactive levers. This difference in responding persisted throughout the first 7 days of short (1-hr) access, and was even more pronounced during the 14 days of long (6-hr) access. After completion of self-administration, rats were further divided into two subgroups and underwent either 14 days of daily extinction training or spent equivalent time in abstinence (Figure 1A). Self-administration data for the abstinent subgroup was previously reported [14], and both sub-groups displayed a similar pattern of meth self-administration. For the extinction group, removal of meth reinforcement produced a rapid decrease of lever pressing in all animals (Figure 1B, right panel).

Both extinction and abstinent rats displayed a similar escalation of daily meth intake. As demonstrated on Figure 1C, meth intake (mg/kg/day) averaged over the last three days of 6-hr access (L12–L14) was significantly higher than average daily intake measured over the first three days of 6-hr access (L1–L3) in both abstinent (t_(13)_ = −3.875, p<0.001) and extinguished subgroups of rats (t_(13)_ = −5.926, p<0.001). This escalation was specific for the extended 6-hr access period and did not occur during the initial 7 days of limited 1-hr access (Figure 1C). In addition, no differences were found between abstinent and extinguished animals in their total cumulative meth intake across the self-administration period (t_(17)_ = −1.764, p = 0.096; Figure 1D).

### Analysis of surface vs. intracellular distribution of mGluR2/3 and mGluR7 receptors in synaptosomes isolated from rat prefrontal cortex and striatum

The majority of previous studies on drug-induced regulation of mGluR receptors have been limited to measurement of total tissue content. The current study employed surface biotinylation techniques in order to measure not only the total number of receptors, but also a pool of surface (presumably active) mGluR2/3 and mGluR7 receptors present in synaptosomal fractions prepared from meth and saline control rats. Figure 2A shows the rat brain atlas coordinates used as guidelines for tissue dissection for preparations of crude synaptosomal fraction. A representative immunoblot analysis of biotinylated synaptosomes isolated from the dorsal striatum (dSTR) is depicted in Figure 2B. We detected a strong signal for both mGluR2/3 and mGluR7 proteins in the dSTR, which corresponds with the fact that both receptors heavily populate glutamatergic afferents into the striatum [35]. We found that the majority of mGluR2/3s or mGluR7s in this brain region were found on the cell surface (B, biotinylated fraction), with only a limited amount present in the intracellular compartment (NB, non-biotinylated fraction). Under the low reducing conditions used in this study (<10 mM DTT), mGluR2/3 and mGluR7 were detected almost exclusively in a dimer form (200–220 kDa), a pattern described previously in both rat and human brain [36], [37]. However, under strongly reducing conditions (100 mM DTT, P2* fraction), both mGluR2/3 and mGluR7 dimers were dissolved and migrated as monomers (∼100–110 kDa; Figure 2B). Since dimerization/oligomerization of G-protein coupled receptors is an essential step in the generation of “mature” functional complexes [38], [39], only the dimeric form of mGluR2/3 and mGluR7 receptors was analyzed in the present study.

Control protein calnexin was found to be present in all fractions, although to a lesser extent in the biotinylated, surface fraction. This fact could be explained either by the ‘normal’ occurrence of calnexin in the plasma membrane [40], [41], or by the presence of broken membrane fragments or leaky synaptosomes in a relatively crude synaptosomal preparation as used in this study (see also [42]). However, prototypical intracellular proteins like ERK1/2 or Ras-like protein RasD1/AGS1 [43] showed only minimal or no presence in the surface (biotinylated) fraction, suggesting that non-specific biotinylation of intracellular proteins was limited (Figure 2B).

### Meth followed by abstinence results in decreased surface and/or total expression of mGluR2/3 in the medial prefrontal cortex and striatum

**Figure 3:**
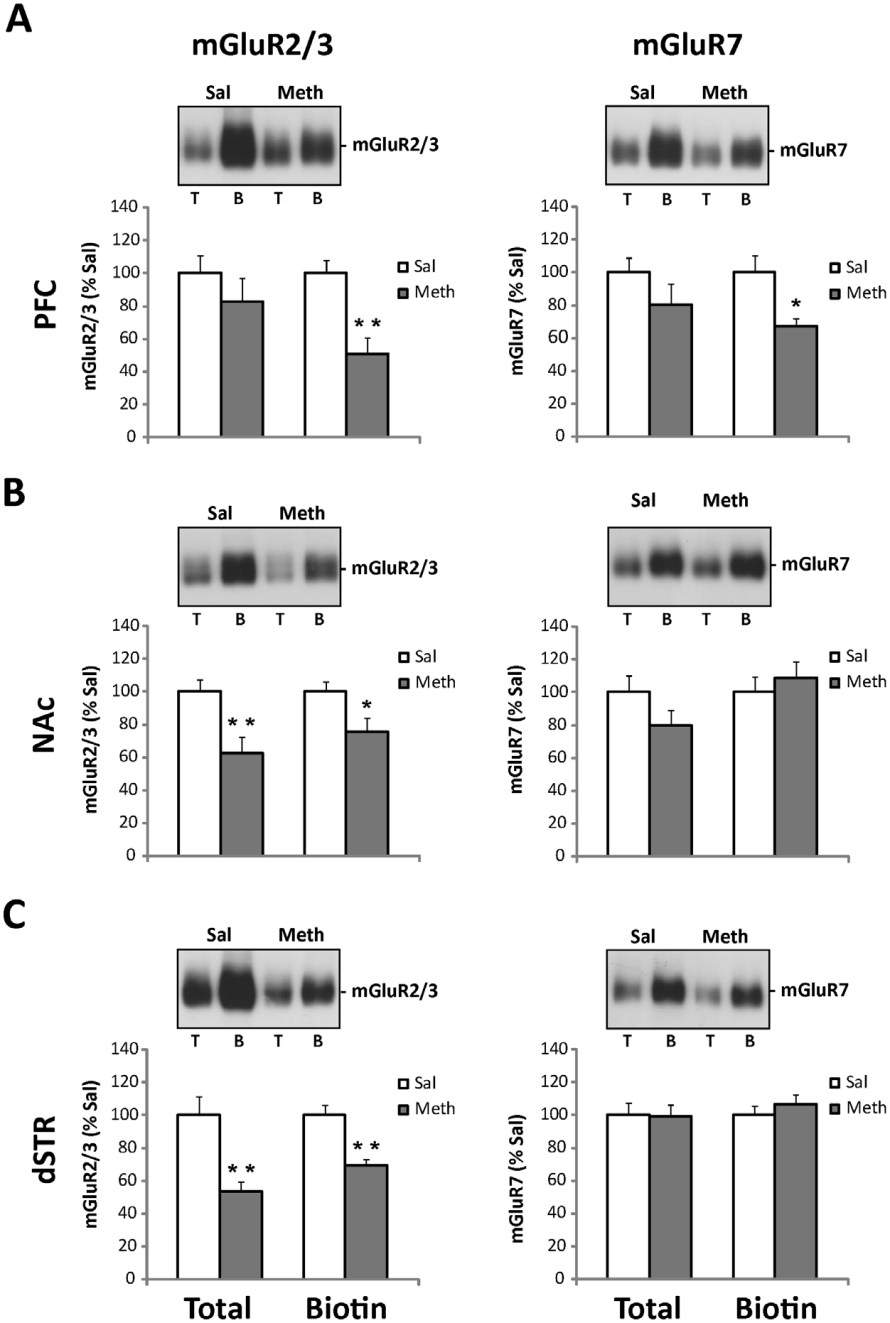
Extended access to intravenous meth followed by abstinence results in decreased surface and/or total expression of mGluR2/3 in the PFC and striatal subregions and decreased surface expression of mGluR7 in the PFC. *Top panels:* Representative immunoblots show levels of mGluR2/3 and mGluR7 protein in total (T) and surface (B, biotinylated) synaptosomal fractions isolated from the PFC (A), NAc (B), and dSTR (C), following meth self-administration and 14 days of abstinence. *Lower panels:* Quantitative analyses of immunoblots revealed significant decreases in total mGluR2/3 levels (B, C), surface mGluR2/3 levels (A, B, C) and surface mGluR7 levels (A) in meth vs. saline rats. Integrated density of each band was analyzed by one-way ANOVA and expressed as the percentage of the saline-treated animals. Data shown as mean ± S.E.M.; n = 11 samples per group. *p<0.05, **p<0.01 vs. Sal.

As demonstrated in Figure 3 (left panels), chronic meth self-administration followed by abstinence resulted in a decreased number of mGluR2/3 receptors present on the surface of synaptosomes (i.e., biotinylated fraction or ‘B’) isolated from the medial prefrontal cortex (PFC; t_(17)_ = 3.69, p<0.01, Figure 3A), nucleus accumbens (NAc; t_(17)_ = 2.37, p<0.05, Figure 3B), and dSTR (t_(20)_ = 4.35, p<0.01, Figure 3C), when compared to mGluR2/3 surface levels in control animals. In addition, total mGluR2/3 receptor levels were also decreased in the NAc (t_(17)_ = 3.05, p<0.01, Figure 3B) and dSTR (t_(20)_ = 3.65, p<0.01, Figure 3C) of meth animals when compared to their saline counterparts. As for mGluR7 (Figure 3, right panels), meth-induced changes were limited to lower surface levels in the PFC (t_(17)_ = 2.80, p<0.05, Figure 3A, right panel).

### Meth followed by daily extinction results only in a decreased surface expression of mGluR2/3 in the medial prefrontal cortex

**Figure 4:**
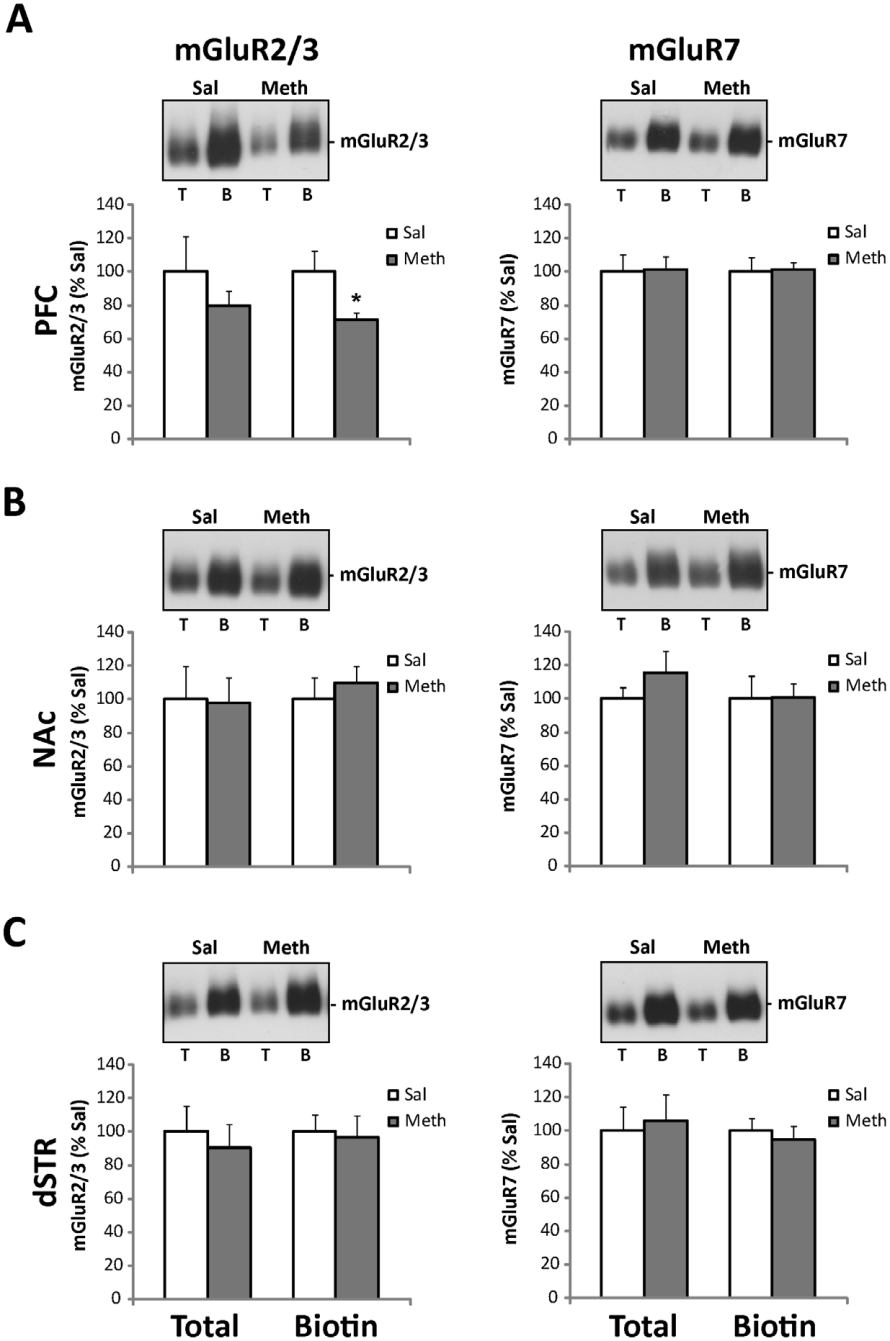
Extended access to intravenous meth followed by extinction results in decreased surface expression of mGluR2/3 in the PFC. *Top panels:* Representative immunoblots show levels of mGluR2/3 and mGluR7 protein in total (T) and surface (B, biotinylated) synaptosomal fractions isolated from the PFC (A), NAc (B), and dSTR (C) following meth self-administration and 14 days of extinction. *Lower panels:* Quantitative analyses of immunoblots revealed significant decreases in surface mGluR2/3 levels in the PFC (A). Integrated density of each band was analyzed by one-way ANOVA and expressed as the percentage of the saline-treated animals. Data shown as mean ± S.E.M.; n = 8 samples per group. *p<0.05 vs. Sal.

In contrast to the multiregional changes in mGluRs seen after abstinence from meth self-administration, chronic meth self-administration followed by daily extinction only resulted in a decreased number of surface mGluR2/3 receptors in synaptosomes isolated from the medial PFC (t_(13)_ = 2.37, p<0.05, Figure 4A, left panel). Changes in total or surface levels of mGluR2/3 (Figure 4, left panels) or mGluR7 (Figure 4, right panels) were not detected in any other analyzed brain regions.

## Discussion

The present study demonstrates that extended access to meth self-administration results in escalated meth intake and persistent alterations in total and/or surface levels of mGluR2/3 and mGluR7 receptors within the corticostriatal circuitry. Importantly, meth-induced changes in mGluR levels are clearly dependent on the conditions of post meth withdrawal. Specifically, daily extinction trials reversed the downregulation of striatal mGluR2/3 receptors found in abstinent rats (who had no extinction experience). In contrast, escalated meth self-administration decreased surface levels of mGluR2/3 receptors in the medial PFC, regardless of post meth experience. These results suggest that extinction learning can selectively restore specific populations of down regulated mGluRs after prolonged exposure to meth. The potential significance of the present findings for the persistence (or the recovery) of meth-induced motivational and cognitive deficits is discussed below.

A large body of evidence from both preclinical and clinical studies suggests that persistent drug seeking and increased risk of relapse are driven by drug-induced neuroadaptations within specific brain circuits of motivation. In animal models, factors contributing to drug seeking have been traditionally studied following extinction, rather than abstinence [44], in order to reduce operant responding prior to reinstatement of drug seeking. While the use of extinction procedures is highly useful for these models, explicit extinction training has been used only infrequently in the treatment of drug addiction [45], [46] and relapse in humans typically occurs after an untreated drug-free period (i.e., abstinence). Extinction training (in contrast to abstinence) involves new learning about the drug-taking environment and as result, drug-seeking after extinction engages brain circuitry differently than that of abstinence (see [47] for review). For example, inactivation of the projections from the dorsomedial PFC (prelimbic cortex) to the NAc (core subregion) blocks the reinstatement of methamphetamine or cocaine seeking in extinguished animals [48], [49], [50]. However, inactivation of dSTR, but not the dorsomedial PFC or NAc inhibits drug seeking in animals following abstinence [32], [51], indicating that extinction training recruits PFC-to-NAc projections in the regulation of drug-seeking. In addition, a dorso-ventral functional divide within the medial PFC has been shown to emerge during extinction training after cocaine self-administration, whereby the dorsomedial PFC (prelimbic cortex) drives cocaine seeking, while the ventromedial PFC (infralimbic cortex) suppresses it [52]. Importantly, such a division does not exist for animals with a history of meth self-administration, in that inactivation of either the dorsal or ventral PFC attenuates meth seeking [50].

Besides engaging different neurocircuitry, extinction training has also been shown to produce glutamatergic neuroadaptations that differ from those found in abstinent animals [31], [53], [54], [55]. Thus, the current study focused on analysis of presynaptic mGluRs within the neurocircuitry that promotes drug-seeking after extinction (dorsomedial PFC and NAc), as well as in the dSTR, which is necessary for drug-seeking after abstinence. In abstinent animals, mGluR2/3 receptors (both total and surface populations) were found to be downregulated in all three regions studied, with mGluR7 being significantly decreased only in the PFC. Interestingly, daily extinction training ‘normalized’ mGluR2/3 levels in the striatum and mGluR7 levels in the PFC. In agreement with these results, extinction may reverse or ameliorate glutamatergic neuroadaptations within the corticostriatal circuitry produced by chronic drug exposure and withdrawal (abstinence), and as a result reduce drug-seeking [56], [57]. mGluR2/3 and mGluR7 are the most abundant presynaptic glutamate autoreceptors found on corticostriatal glutamatergic terminals [58], [59] and activation of either mGluR2/3 or mGluR7 inhibits drug-seeking through inhibition of corticostriatal glutamate transmission during reinstatement [60], [61]. Therefore, a decrease in mGluR2/3 autoreceptor function (such as decreased surface expression of mGluR2/3 receptors described in the current study) may contribute to increased releasability of glutamate in the striatum and enhanced reinstatement of drug seeking [27], [62]. Indeed, prolonged meth self-administration followed by abstinence resulted in a robust reinstatement [63] and sensitized response of NAc glutamate to a meth-priming injection [20]. Interestingly, the ability of modafinil, a putative mGluR2/3 agonist [64], to block reinstatement of meth seeking is also attenuated in abstinent animals when compared to their extinguished counterparts [63]. These findings further support the possibility that corticostriatal mGluR2/3 receptor function is decreased after abstinence from chronic meth, but can be recovered with extinction training.

Different brain region-specific cellular mechanisms could be implicated in regulation of mGluR2/3 and mGluR7 receptors reported in the present study. Since mGluR2/3 and mGluR7 in the striatum (NAc and dSTR) are almost exclusively located within axon terminals [58], [59], simultaneous downregulation and recovery of total and surface receptor levels could be a consequence of both local (striatal) and extra-striatal regulatory mechanisms. For example, decreased availability of striatal mGluR2/3 receptors in abstinent meth rats could arise from disrupted receptor membrane trafficking at the striatal synapse, as well as from decreased protein synthesis and anterograde delivery of mGluR2/3s from striatal input regions. The relative contribution of each mechanism will need to be addressed in future studies. On the other hand, surface (but not total) levels of mGluR2/3 and mGluR7 receptors were altered in the medial PFC, indicating specific changes in mechanisms regulating receptor surface trafficking. Extinction-dependent regulation of mGluR5 receptor trafficking has been recently reported [53]; however, the current data are the first to suggest chronic psychostimulant-induced changes in mGluR2/3 and mGluR7 receptor trafficking in glutamatergic presynaptic terminals. It should be noted that in the present study and other previous studies using crude synaptosomal preparations, a general assumption is that the analysis reflects changes in surface trafficking within nerve terminals. However, this type of preparation is not completely devoid of glial membrane fragments [65]. Since functional mGluR2/3 receptors have been described in astrocytes [66], it cannot be ruled out that meth self-administration and post-meth experience alters mGluR2/3 levels in non-neuronal membranes.

Reduced levels of mGluR2/3 and mGluR7 receptors observed in the striatum (and PFC) after high doses of meth may have potentially been caused by neuronal damage or glial activation as a result of neurotoxicity [67]. However, this is unlikely as: a) meth-induced decreases in presynaptic mGluRs were reversed by extinction training, and b) we have previously shown that this particular extended access meth self-administration regimen does not produce lasting neurotoxicity in the forebrain as markers for dopamine terminals, astrocytes, and microglia were not altered at 7–14 days after meth self-administration [13], [68].

Previous findings from our laboratory and others have demonstrated that rats with extended access meth self-administration display both enhanced drug seeking [12], [13], as well as deficits in cognitive and memory domains [12], [14], [68]. These deficits persist at least up to 14 days after the last meth self-administration session [12], [14], [68], which correlates with our findings of dysregulated mGluR levels at this time point (current study). Disrupted mGluR2/3 and/or mGluR7 receptor function in the forebrain has also been implicated in deficits in cognitive processing often diagnosed in patients with schizophrenia [69], with findings from several preclinical models of schizophrenia as well as from postmortem studies showing decreased levels of mGluR2/3 receptors, predominately in the PFC [70], [71]. Since chronic meth exposure has been used to model some schizophrenia symptoms in experimental animals [72], [73], it is possible that meth–induced memory and cognitive deficits could also be attributed to altered mGluR2/3 function. In particular, since the deficits have been previously detected in animals regardless of extinction training [12], [14], disrupted surface trafficking of mGluR2/3 in the PFC could represent a critical neurobiological meth-induced deficit, independent of post-meth experience. The exact neurobiological mechanisms by which meth self-administration alters homeostasis of mGluR2/3 receptor trafficking remains unknown and will be pursued in future experiments.

In conclusion, the current study presents novel findings on potential substrates of motivational and cognitive deficits found in animals after extended access to meth self-administration. Specifically, meth produces a prolonged downregulation of mGluR2/3 and mGluR7 surface expression within the corticostriatal circuitry of abstinent animals, an effect that can be reversed (at least in the striatal glutamatergic terminals) by daily extinction during withdrawal. While future studies will need to directly assess the behavioral significance of altered corticostriatal mGluR2/3 and mGluR7 receptors, these findings are of particular significance as recent evidence suggests that agents which positively modulate function of mGluR2/3 receptors (e.g., modafinil, [64]) also inhibit meth seeking [63], [74] and may possess therapeutic potential both for the prevention of relapse and improving cognitive deficits in meth-dependent individuals [75], [76].
